# Trends and projections of the burden of disease for male infertility in China from 1990 to 2021: an analysis from the Global Burden of Disease 2021 study

**DOI:** 10.3389/frph.2024.1501675

**Published:** 2024-12-16

**Authors:** Ran Xu, Xin-jun Wang, Qing-cheng Lin, Yan-ting Zhuang, Qing-ying Zhou, Nai-fen Xu, Ding-qin Zheng

**Affiliations:** ^1^ Department of Urology, Pingyang Hospital of Wenzhou Medical University, Wenzhou, China; ^2^ Department of Urology, Zhongshan Hospital Xiamen University, School of Medicine, Xiamen University, Xiamen, China; ^3^ Department of Xiaojiang, Pingyang Hospital of Wenzhou Medical University, Wenzhou, China; ^4^ Department of Hepatobiliary and Pancreatic Surgery, Zhongshan Hospital Xiamen University, School of Medicine, Xiamen University, Xiamen, China

**Keywords:** male infertility, Global Burden of Disease, Joinpoint regression, age-period-cohort analysis, prevalence

## Abstract

**Background:**

Currently, male infertility represents a serious disease burden worldwide, and China is one of the most affected countries. The aim of this study was to examine the evolution of the disease burden of male infertility in China during the period 1990–2021 and to project the trend for 2022–2036.

**Methods:**

By screening and processing data from the Global Burden of Disease 2021, this study obtained data on the prevalence, disability-adjusted life years and corresponding rates and age-standardised rates of male infertility between 1990 and 2021. To assess the trend in the burden of male infertility over the past 30 years, the annual percentage change and the average annual percentage change were calculated from the above data using Joinpoint regression models. In addition, age-period-cohort models were used to estimate the independent effects of age, period and cohort factors on male infertility, and Bayesian projection models were used to predict the trend in the disease over the next 15 years.

**Result:**

In both 1990 and 2021, the burden of male infertility tended to increase and then decrease with age, with the heaviest burden in the 35–39 age group. Meanwhile, the Joinpoint model found statistically significant average annual percentage changes in age-standardised prevalence and age-standardised disability-adjusted life years of 0.14% and 0.19%, respectively. In addition, the trend for both was a gradual increase over time until 1994 and a gradual decrease over time after 1994. In the age-period-cohort analysis model, age, period and cohort effects indicated that 35–39 years, 1997–2001 and 1945–1949 were the years with the highest risk of male infertility. Finally, the Bayesian projection model suggested that the disease burden of male infertility in China would show a decreasing trend over the next 15 years.

**Conclusion:**

From 1990 to 2021, the disease burden of male infertility in China generally increased. However, thanks to a number of factors, including China's proactive health policies and effective management, the burden of disease has continued to decrease in the last decade and is projected to continue declining from 2022 to 2036. To sustain this positive trend, it remains essential for China to maintain and strengthen effective management and control of male infertility.

## 1 Introduction

The World Health Organization defines infertility as the failure of a couple to achieve a natural pregnancy after one year of regular, unprotected intercourse (1). Epidemiological studies have shown that infertility affects approximately 8%–12% of couples of appropriate age worldwide (2), and that up to 20% of these cases are caused by male factors alone and 30% by a combination of male and female factors (3, 4). In addition, previous studies have shown that the age-standardised prevalence rate of male infertility worldwide increased at an annual rate of 0.29% between 1990 and 2017 (5).

Not only does male infertility impose a serious psychological burden on the patient, but in recent years male infertility has been increasingly associated with a number of chronic diseases, including tumours, cardiovascular disease, autoimmune diseases and many other chronic conditions (6–9). On the other hand, at a macro societal level, the disease burden of male infertility can lead to a number of difficulties such as declining fertility rates, ageing populations, labour shortages, lack of socio-economic vitality and increased pressure on welfare and security systems (10).

Over the past two decades, China's influence in economic growth, technological innovation and international affairs has grown. Today, as the world's second largest economy, China has risen significantly on the international stage and plays an increasingly important role in international organisations. At the same time, China's demographic structure has changed significantly over this period, with a declining birth rate and an ageing population (11). There are many reasons for this phenomenon, and male infertility plays an important role.

Huang et al. conducted a comprehensive study of the global burden of male infertility from 1990 to 2019 (12). In this study, China ranked first in the world in the prevalence of male infertility in 2019, with 14.57 million cases. To provide up-to-date and valuable information for the management of male infertility in China, we used data from the recently updated Global Burden of Disease (GBD) database to conduct an epidemiological assessment of the burden of male infertility in China and a systematic projection of the evolution of the burden of male infertility in China from 2022 to 2036.

## 2 Methods

### 2.1 Data resources and definitions

In this study, data on the burden of male infertility were obtained from the GBD database, which was initiated and compiled by organizations such as the World Bank and the World Health Organization to estimate the burden of a wide range of diseases and injuries on a global scale. The GBD2021 database, which includes a broad and representative range of data sources, contains data on 371 diseases and injuries and 88 major risk factors from 204 countries and territories. Additionally, GBD2021 includes data spanning from 1990 to the present, enabling researchers to effectively assess long-term trends and changes in the burden of disease through a retrospective approach.

GBD data are generated from a combination of direct and indirect sources (e.g., national health surveys, statistical modeling, and expert opinion) to estimate the burden of disease, particularly in areas with limited data. The sampling methodology of the GBD database varies based on the data available in each country or region. For countries with established vital registration systems, mortality and morbidity data are reported directly. In regions where such data are unavailable, statistical modeling is used to estimate disease burden, integrating data from household surveys, hospital records, and expert input.

Regarding inclusion and exclusion criteria, the GBD database encompasses a wide range of conditions, including male infertility, classified according to the International Classification of Diseases (ICD) coding system. Male infertility is assigned the ICD-11 code GB04, which includes the subcategories of azoospermia (GB04.0), other specified male infertility (GB04.Y), and unspecified male infertility (GB04.Z). Additionally, drug-induced male infertility due to testicular hypoplasia is classified under 5A81.1, and male infertility due to cystic fibrosis is classified under CA25.0, CA25.1, and CA25.Z. It is important to note that the GBD database does not collect clinical diagnoses directly but compiles epidemiological data based on reports from healthcare providers.

### 2.2 Descriptive analysis

In this study, we used prevalence number, prevalence rate, age-standardised prevalence rate (ASPR), disability-adjusted life years (DALYs), DALYs rate and age-standardised disability-adjusted life years rate (ASDR) to assess, describe and predict the trend of the burden of male infertility in China. DALYs are defined and explained on the official GBD database website as follows: “DALY is an abbreviation for disability-adjusted life year. It is a universal metric that allows researchers and policymakers to compare very different populations and health conditions across time. One DALY equals one lost year of healthy life. DALYs allow us to estimate the total number of years lost due to specific causes and risk factors at the country, regional, and global levels.”

### 2.3 Analysis of temporal trends

In this study, a time trend analysis of the disease burden of male infertility in China was conducted using the Joinpoint regression model, which analyses the trend change points and identifies the join points in the time series data, and then decomposes the overall trend into several sub-trends (13–15). As a statistical method for analysing local disease trends, the Joinpoint regression model is more suitable for public health and epidemiological studies than other methods for analysing overall disease trends. The results of the Joinpoint regression model are expressed as annual percent change (APC) and average annual percent change (AAPC). An APC or AAPC greater than 0 indicates that the burden of disease has tended to increase over time during the period; conversely, that the burden of disease has tended to decrease. *p*-values less than 0.05 were considered statistically significant.

### 2.4 Age-period-cohort analysis and projection

The age-period-cohort (APC) model is a statistical tool used in epidemiology, demography, and the social sciences to analyze the effects of three distinct time-related factors on specific outcomes or trends: the age effect, the period effect, and the cohort effect (16, 17). The age effect refers to the impact of biological aging on outcomes; the period effect captures simultaneous influences on all age groups during a specific time period, reflecting societal or environmental changes; and the cohort effect represents variations in outcomes among groups born in different generations due to differing exposures or conditions (18).

In this study, we conducted an APC analysis of the disease burden of male infertility in China from 1990 to 2021. This analysis was performed by establishing a reference group for the APC model (15–19 years for age, 1992–1996 for period, and the cohort born in 1977) and importing the processed GBD data into an online tool (https://analysistools.cancer.gov/apc/) (19). These reference categories were selected for their central position within the dataset and their relatively stable estimated effects, providing a balanced and representative baseline for comparison.

In addition, to better manage male infertility in China in the future, we used Bayesian age-period-cohort (BAPC) models to project the evolution of the disease burden of male infertility in China between 2022 and 2036. The BAPC model combines the advantages of Bayesian statistical methods and can be used to analyse and project the effects of age, period and birth cohort on an event in demographic data, and is able to better account for the linear dependence between the three effects compared with the traditional APC model (20). In addition, the selection of 2036 as the projection endpoint is based on the standard projection horizon used in the GBD database for long-term forecasts. Projections are typically made for a 15-year period following the most recent data, which in this case is 2021. Thus, the year 2036 represents a reasonable point for forecasting future trends based on current data and assumptions about future changes in healthcare and population demographics.

### 2.5 Statistical metrics

All of the above analyses were primarily performed using R software (version 4.2.3). The statistical results of this study were presented using the Uncertainty Interval (UI), Confidence Interval (CI), and Relative Risk (RR).

As defined by the GBD website, the Uncertainty Interval (UI) is “a range of values that reflects the certainty of an estimate.” In GBD, every estimate is calculated 1,000 times, with each iteration sampling from distributions rather than using point estimates for data inputs, transformations, and model choices. The 95% UI is determined by the 25th and 975th percentiles of these 1,000 ordered estimates. Larger uncertainty intervals may result from limited data availability, small studies, or conflicting data, while smaller uncertainty intervals typically arise from extensive data, large studies, and consistent sources. In addition to the UI, this study also calculates the Relative Risk (RR) of male infertility based on prevalence rates across different time periods and population groups. The RR compares the risk of male infertility in various groups relative to a reference group. Moreover, the Confidence Interval (CI), a statistical range reflecting the uncertainty around the estimate of a parameter, is used to quantify the precision of our estimates. In this study, 95% CIs are reported to represent the uncertainty surrounding the estimated ASPR and other key metrics.

## 3 Results

### 3.1 Disease burden of male infertility in 2021 compared to 1990

**Figure 1:**
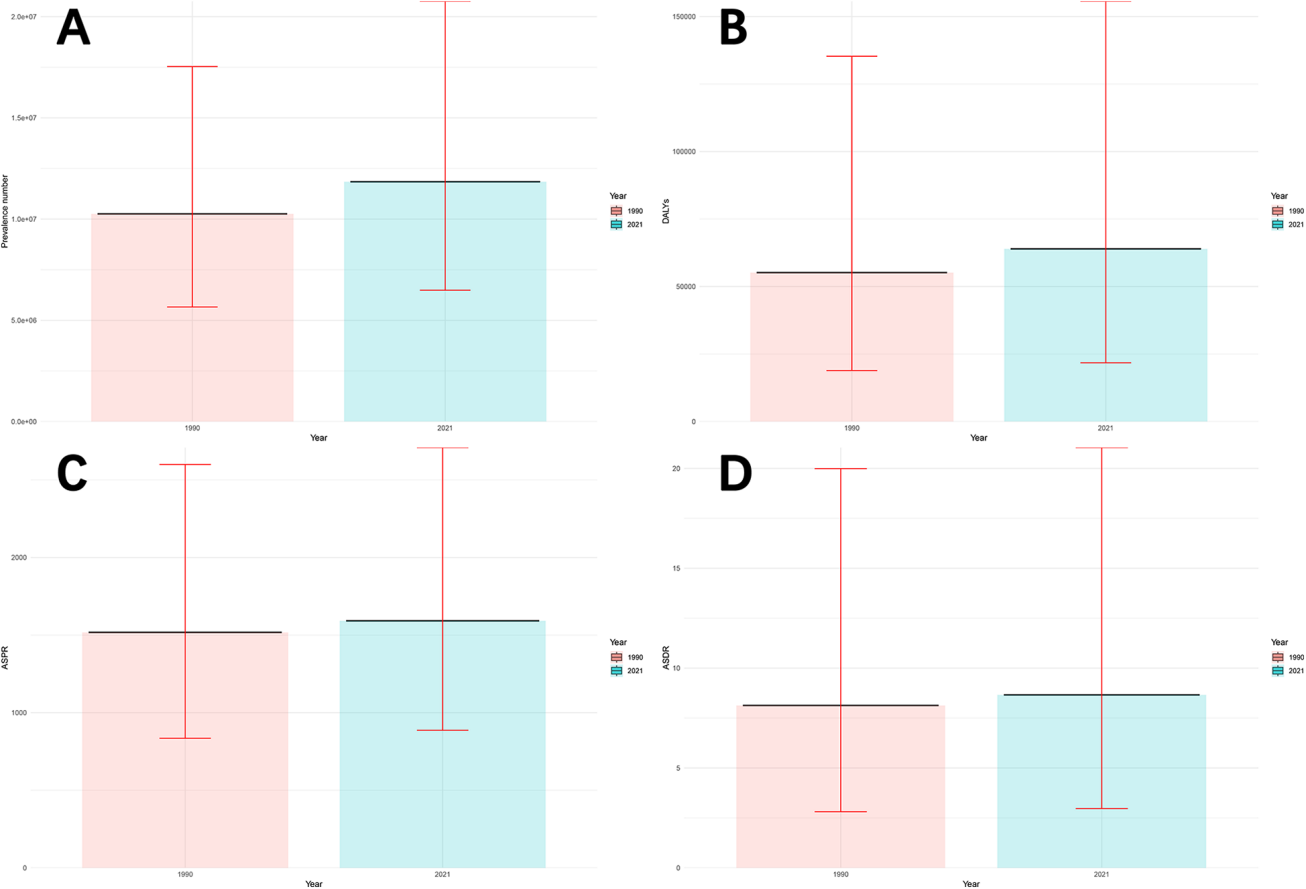
Disease burden of male infertility in China in 1990 and 2021. **(A)** Number of prevalence cases (in millions), with 95% uncertainty intervals (UI). **(B)** Disability-Adjusted Life Years (DALYs) (in thousands), with 95% uncertainty intervals (UI). **(C)** Age-Standardized Prevalence Rate (ASPR) (per 100,000 population), with 95% uncertainty intervals (UI). **(D)** Age-Standardized Death Rate (ASDR) (per 100,000 population), with 95% uncertainty intervals (UI). Prevalence cases, DALYs, ASPR, and ASDR in 1990 and 2021 are compared, showing changes over time with associated uncertainty intervals.

Figure 1 and Supplementary Table S1 show the disease burden of male infertility in China in 1990 and 2021. Briefly, the number of prevalence cases increased from 10.26 million (95% UI: 5.66–17.53 million) in 1990 to 11.85 million (95% UI: 6.49–20.76 million) in 2021, an increase of 15.49%. The ASPR increased from 1,517.39 per 100,000 people (95% UI: 834.93–2,599.79) in 1990 to 1,591.79 per 100,000 people (95% UI: 886.51–2,708.22) in 2021, an increase of 4.90%. In addition, DALYs increased from 55.16 thousand (95% UI: 18.88 to 135.27 thousand) in 1990 to 63.93 thousand (95% UI: 21.75–155.61 thousand) in 2021, an increase of 15.76%. The ASDR increased from 8.13 per 100,000 people (95% UI: 2.81–19.99) in 1990 to 8.66 per 100,000 people (95% UI: 2.97–21.04) in 2021, an increase of 6.52%.

**Figure 2:**
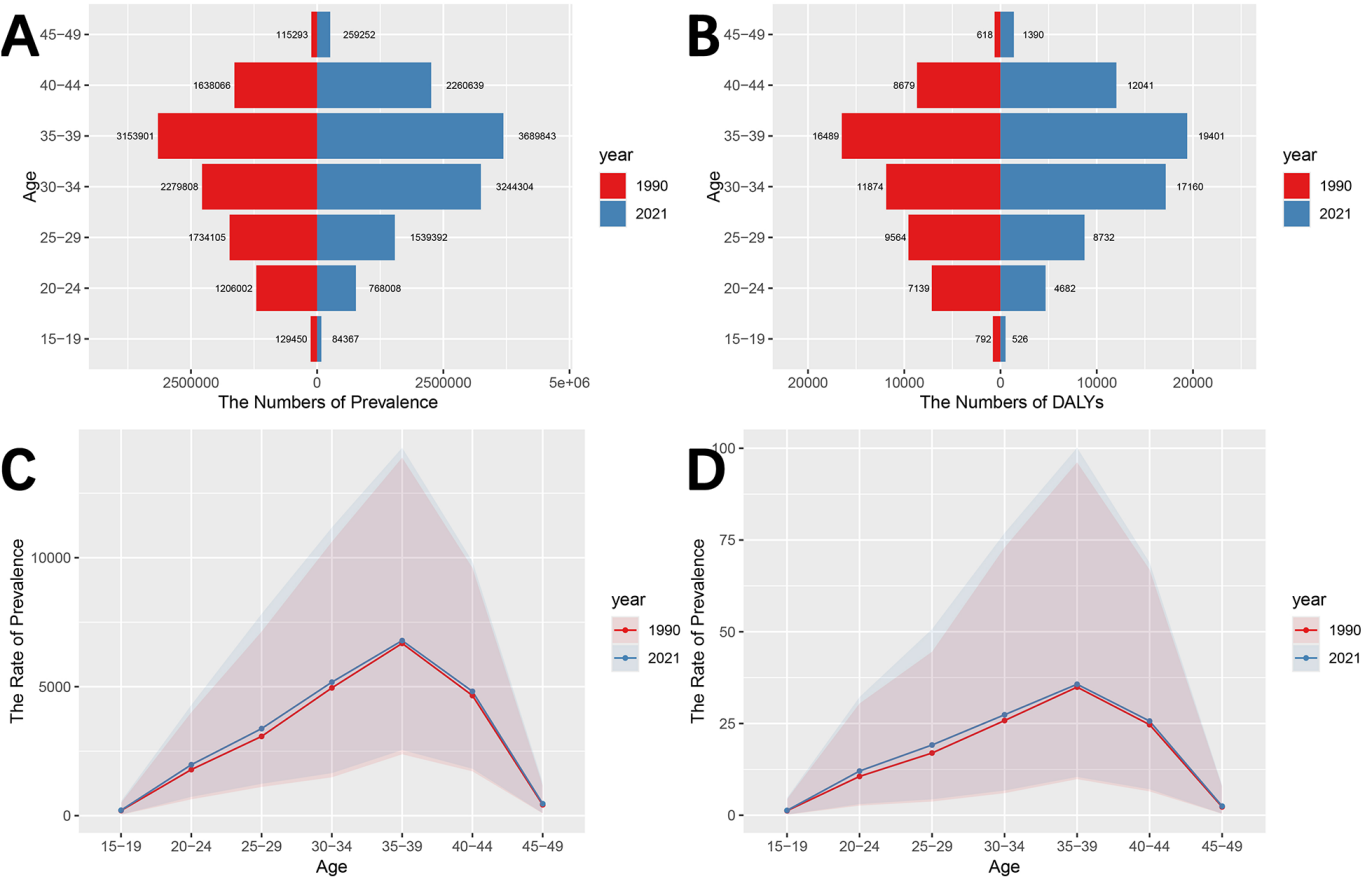
Disease burden of male infertility in China by age groups in 1990 and 2021. **(A)** Number of prevalence cases by age group in 1990 and 2021. **(B)** DALYs by age group in 1990 and 2021. **(C)** Prevalence rate (per 100,000 population) by age group in 1990 and 2021. **(D)** DALYs rate (per 100,000 population) by age group in 1990 and 2021. Trends in male infertility burden (prevalence cases, DALYs, prevalence rates, and DALYs rates) are shown for 1990 and 2021. The data indicate an increase in prevalence rates and DALYs in the 35–39 age group, and a trend towards older ages affected by male infertility.

The disease burden of male infertility at different ages in China in 1990 and 2021 is shown in Figures 2A,B and Supplementary Table S2. First, in both 1990 and 2021, the number of prevalence cases showed an increasing and then decreasing trend with age, with the highest number of cases occurring in the 35–39 age group. The number of prevalence cases in each age group as a percentage of total prevalence cases in 1990 was: 1.26% (15–19 years), 11.76% (20–24 years), 16.91% (25–29 years), 22.22% (30–34 years), 30.75% (35–39 years), 15.97% (40–44 years) and 1.12% (45–49 years). In 2021, the percentage of prevalence cases in each age group was 0.71% (15–19 years), 6.48% (20–24 years), 13.00% (25–29 years), 27.39% (30–34 years), 31.15% (35–39 years), 19.00% (40–44 years) and 2.19% (45–49 years). It is clear that the average age of male infertility is higher in 2021 than in 1990.

Similar to the number of prevalent cases, the number of person-years of DALYs tends to increase and then decrease with age, peaking at 35–39 years. Among these, the percentage of DALYs in each age group in the total population in 1990 was: 1.43% (15–19 years), 12.94% (20–24 years), 17.34% (25–29 years), 21.53% (30–34 years), 29.90% (35–39 years), 15.74% (40–44 years) and 1.12% (45–49 years). The percentage of DALYs in each age group in 2021 is: 0.82% (15–19 years), 7.32% (20–24 years), 13.66% (25–29 years), 26.84% (30–34 years), 30.35% (35–39 years), 18.83% (40–44 years) and 2.17% (45–49 years). In addition, the largest increase in DALYs occurred in the 30–34 age group (5.31%), and the largest decrease in DALYs occurred in the 20–24 age group (5.62%).

As can be seen in Figures 2C,D and Supplementary Table S2, the prevalence rates and DALYs rates of male infertility follow a similar trend to the number of prevalence cases and DALYs, both of which increase and then decrease with age, reaching the highest risk level in the 35–39 age group. The difference is that the prevalence rates and DALYs rates in the 15–19, 20–24 and 25–29 age groups are higher in 2021 than in 1990, while the number of prevalence cases and DALYs are lower in 2021 than in 1990.

### 3.2 Analysis of the Joinpoint regression models

**Figure 3:**
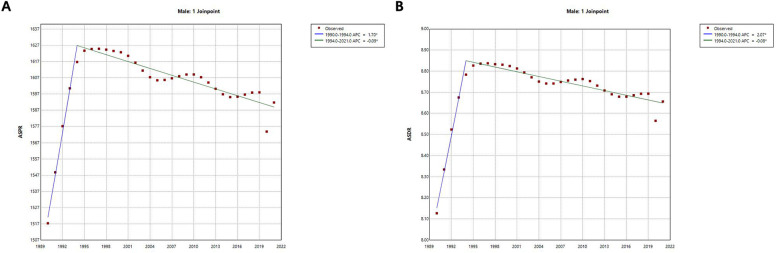
Joinpoint regression analysis of trends in ASPR and ASDR for male infertility in China from 1990 to 2021. **(A)** Age-Standardized Prevalence Rate (ASPR) with Joinpoint regression and associated annual percentage change (APC) and average annual percentage change (AAPC). **(B)** Age-Standardized Death Rate (ASDR) with Joinpoint regression and associated annual percentage change (APC) and average annual percentage change (AAPC). Joinpoint regression models show trends in ASPR and ASDR for male infertility over time. Significant changes in both ASPR and ASDR are observed, with a rapid increase until 1994 followed by a gradual decline thereafter. Annual percentage change (APC) and average annual percentage change (AAPC) values for both rates were calculated, with statistical significance (*p* < 0.001).

Joinpoint regression analysis shows (Figure 3 and Table 1) that the ASPR increases overall from 1990 to 2021, with an AAPC of 0.14%. In terms of sub-trends, the ASPR continues to increase significantly in the period 1990–1994, followed by a gradual and slow decline in the period 1995–2021, and the APC is 1.70% and −0.09% for the periods 1990–1994 and 1995–2021, respectively. On the other hand, the trend of the ASDR is very similar to that of the ASPR, which also shows a significant increase until 1994 and a gradual and slow decrease in 1995 and thereafter. The ASDR has an AAPC of 0.19% for the period 1990–2021 and an APC of 2.07% and −0.09% for the periods 1990–1994 and 1995–2021 respectively. Finally, both the AAPC and APC values for ASPR and ASDR were statistically significant (*p* < 0.001).

**Table 1:** Joinpoint regression analysis: APC and AAPC of ASPR and ASDR for male infertility in China from 1990 to 2021.

	Year	APC/AAPC (%)	95% CI	Test statistic (*t*)	*p*-Value
ASPR	1990–1994	1.70	1.42–1.97	12.68	<0.001
	1994–2021	−0.09	−0.10 to −0.07	−12.14	<0.001
	1990–2021	0.14	0.11–0.18	7.73	<0.001
ASDR	1990–1994	2.07	1.80–2.35	15.79	<0.001
	1994–2021	−0.09	−0.10 to −0.07	−11.95	<0.001
	1990–2021	0.19	0.16–0.23	10.68	<0.001

AAPC, average annual percent change presented for full period; APC, annual percent change; CI, confidence interval; ASPR, age-standardized prevalence rate; ASDR, age-standardized disability adjusted life year rate.

### 3.3 Analysis of APC models

**Figure 4:**
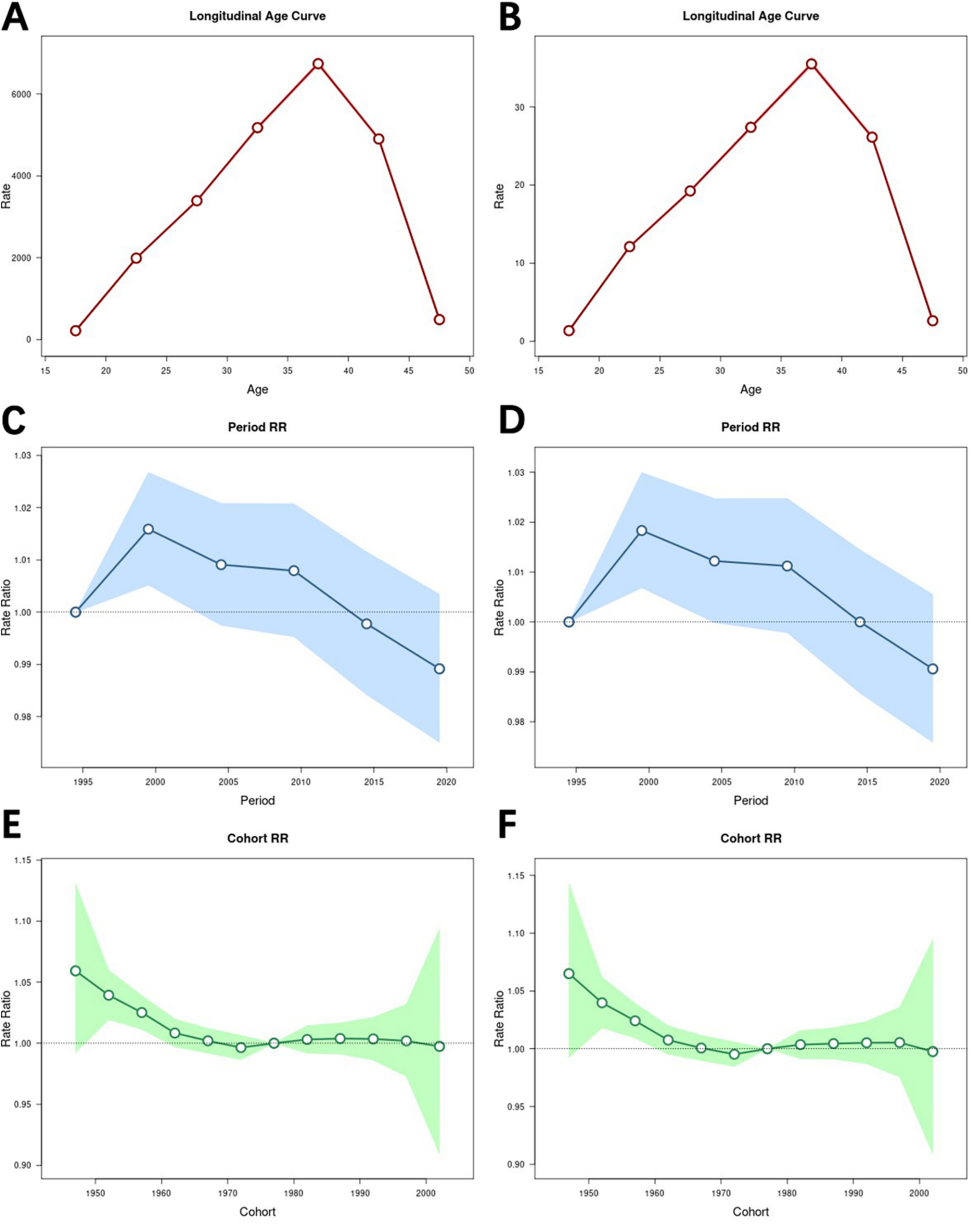
Impact of age, period, and cohort effects on the burden of male infertility. **(A)** Age effect on the prevalence rate, with relative risk (RR) and 95% confidence intervals (CI) for different age groups. **(B)** Age effect on the rate of DALYs, with relative risk (RR) and 95% confidence intervals (CI) for different age groups. **(C)** Period effect on the prevalence rate, with relative risk (RR) and 95% confidence intervals (CI) for different periods. **(D)** Period effect on the rate of DALYs, with relative risk (RR) and 95% confidence intervals (CI) for different periods. **(E)** Cohort effect on the prevalence rate, with relative risk (RR) and 95% confidence intervals (CI) for different birth cohorts. **(F)** Cohort effect on the rate of DALYs, with relative risk (RR) and 95% confidence intervals (CI) for different birth cohorts. Age-period-cohort models reveal that the burden of male infertility varies by age, period, and cohort. The 35–39 age group had the highest prevalence rate and DALYs, while the 1997–2001 period and 1945–1949 cohort showed the highest risk levels.

The results of the age effect analysis (Figures 4A,B) showed that the prevalence rate initially increased with age, reaching its highest level in the 35–39 year age group (RR = 6,741.01; 95% CI = 6,682.51–6,800.02), and then continued to decrease with age. Similarly, the rate of DALYs increased until the age of 35–39 years and then showed a decreasing trend with increasing age, with a risk of (RR = 35.51; 95% CI = 35.18–35.85) in the age group 35–39 years.

The results of the period effect analysis (Figures 4C,D) showed that both the prevalence rate and the rate of DALYs tended to increase and then decrease over time, and that both the prevalence rate (RR = 1.02; 95% CI = 1.01–1.03) and the rate of DALYs (RR = 1.02; 95% CI = 1.01–1.03) were highest in the period 1997–2001.

The results of the cohort analyses (Figures 4E,F) showed that the prevalence rate and the rate of DALYs generally decreased over time. Furthermore, both the prevalence rate and the rate of DALYs peaked in the 1945–1949 cohort, with RR = 1.06 (95% CI = 0.99–1.13) for the prevalence rate and RR = 1.07 (95% CI = 0.99–1.14) for the rate of DALYs. Moreover, both the prevalence rate and the rate of DALYs were lowest in the 1970–1974 cohort, with RR = 1.00 (95% CI = 0.99–1.01) for the prevalence rate and RR = 1.00 (95% CI = 0.99–1.01) for the rate of DALYs.

### 3.4 Predictive analysis of BAPC models

**Figure 5:**
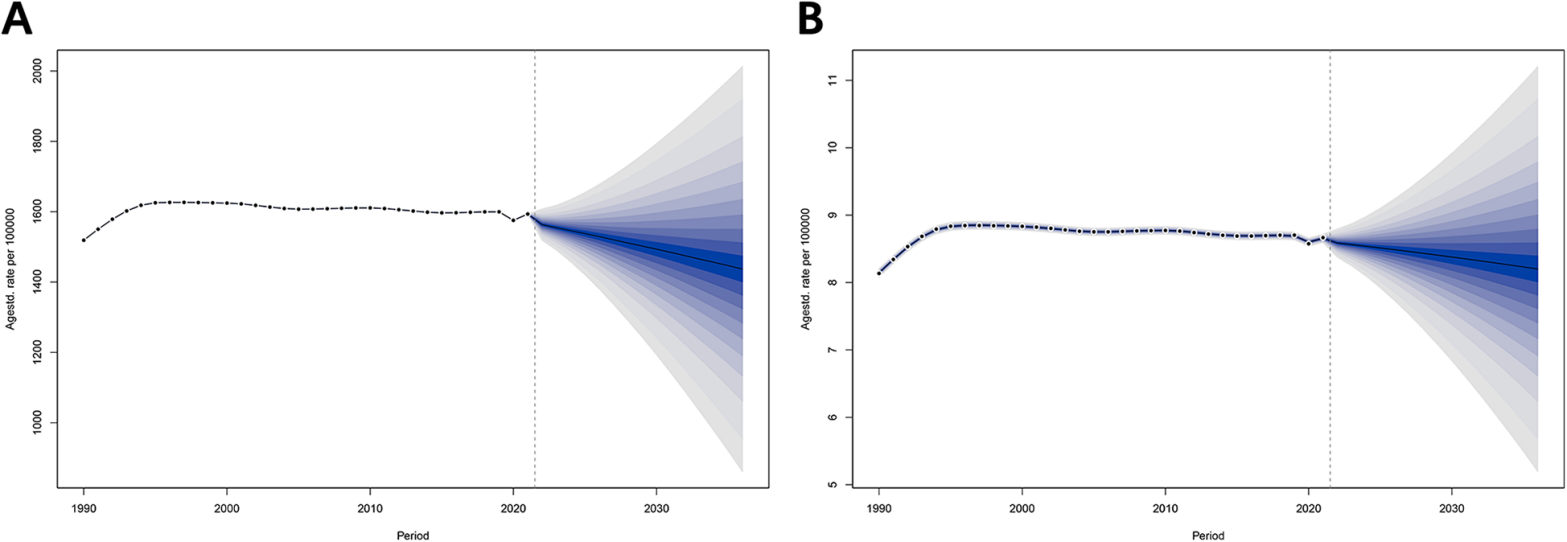
Projected disease burden of male infertility in China, 2022–2036. **(A)** Projected Age-Standardized Prevalence Rate (ASPR) for 2022–2036. **(B)** Projected Age-Standardized Death Rate (ASDR) for 2022–2036. Bayesian prediction models suggest a sustained decrease in ASPR and ASDR for male infertility from 2022 to 2036. Projections include 95% credible intervals to indicate uncertainty in future trends.

According to the Bayesian prediction model (Figure 5 and Supplementary Table 5), the ASPR and ASDR for male infertility will show a similar and sustained downward trend from 2022 to 2036. Specifically, the ASPR is projected to decrease from 1,563.24 per 100,000 people (95% CI = 1,518.95–1,607.54) in 2022 to 1,436.67 per 100,000 people (95% CI = 868.27–2,005.07) in 2036, while the ASDR is projected to decrease from 8.59 per 100,000 people (95% CI = 8.38–8.80) in 2022 to 8.20 per 100,000 people (95% CI = 5.20–11.20) in 2036.

## 4 Discussion

Given the ageing population in China (21), the management of male infertility is of great importance. In this study, we screened and processed relevant data from the GBD database and conducted a comprehensive analysis of the long-term disease burden of male infertility in China using the Joinpoint regression model, the APC model and the BAPC prediction model. The results will help in the management and treatment of male infertility in China.

The study found that the number of prevalence cases, ASPR, DALYs and ASDR of male infertility in China in 2021 will increase more than in 1990. For different age groups, both the number of prevalent cases and DALYs in 1990 and 2021 showed a trend of increasing and then decreasing with age, and the age group with the highest number was in the range of 35–39 years. This may be because, as men age, they are exposed for longer to adverse lifestyle factors such as smoking, alcohol consumption, obesity and psychological stress (22–28), which have long been shown to affect male infertility to varying degrees. In addition to this, environmental or occupational factors (e.g., exposure to toxic chemicals) may also have a gradual potential impact as we age (29). According to this argument, the burden of male infertility should increase with age, but in fact it peaks between the ages of 35 and 39 and then declines rapidly. The reason for this may be that the fertility intentions of the male population in general decline significantly after the age of 40, which in turn may lead to a significant reduction in the detection of male infertility through preconception screening (30). Furthermore, reproductive health programmes do not form part of routine health care, and older men have lower uptake of reproductive health care.

Comparing the 2021 data with the 1990 data, it is easy to see that the number of prevalence cases and DALYs in the 15–19, 20–24 and 25–29 age groups are significantly lower in 2021 than in 1990. Paradoxically, however, the ASPR and ASDR in 2021 are still higher than in 1990 for the same three age groups, which may be due to changes in China's demographic structure in the context of a prolonged period of low fertility (31). Therefore, the fight against male infertility among young people still has a long way to go.

Analysing the data from the Joinpoint regression model, it is easy to see that the turning point in the disease burden of male infertility in China occurred in 1994. We see several possibilities for the rapid deterioration of the disease burden between 1990 and 1994 and the gradual improvement between 1995 and 2021. First, China's rapid industrialisation in the early 1990s led to an increase in environmental pollution (32, 33). Studies have shown that exposure to heavy metals, pesticides and chemicals is strongly associated with reduced male fertility (34, 35). In contrast, after 1994, as the government began to implement environmental protection policies (36–38), pollution levels gradually decreased, which may have improved male fertility. Secondly, with economic growth and changes in public health policy (39–41), testing and treatment for male infertility has become more widespread. This is despite the fact that increased public awareness of infertility has increased the proportion of early medical consultations, leading to an increase in the initial statistics. However, as medical facilities and technology have improved (31, 42), male infertility can be treated more effectively and at an earlier stage, reversing the deterioration of the condition. Thirdly, with the rapid development of Chinese society and increasing public health awareness (43, 44), there has been a gradual improvement in men's lifestyles, such as smoking, alcohol consumption and dietary patterns, which has had a positive impact on fertility.

The APC model analyzes the burden of disease in three directions: age, period, and cohort effects. The results of the analysis of the age effect are very consistent with the previous conclusion that the risk of disease burden is highest at the age of 35–39 years, demonstrating the reliability of the results.

On the other hand, in terms of period effects, the burden of disease peaked around 2,000 and then declined in the APC model, whereas the inflection point of the change occurred around 1994 in the Joinpoint model. This difference is likely because the Joinpoint model captured significant changes in a specific period, identifying abrupt shifts in trends. In contrast, the APC model focuses on long-term trends and structural changes, integrating gradual, cumulative effects over time (15, 16). The APC model is better suited for detecting broader societal shifts, such as the cumulative impact of environmental policies, healthcare improvements, and increased public awareness over several years. This difference in model behavior may be due to the distinct sensitivities and analytical focus of the two models. The Joinpoint regression model is particularly sensitive to specific, statistically significant changes over shorter periods, making it ideal for identifying immediate turning points. In contrast, the APC model decomposes the data into long-term effects (e.g., period and cohort trends), which can smooth out short-term fluctuations but provide a more comprehensive picture of how societal changes influence disease burden over time. Finally, the cohort effect fully demonstrated an overall downward trend in the risk of male infertility in each birth cohort, which may be related to improved standards of care and the strong development of preventive and curative measures.

The BAPC trend prediction model shows a positive downward trend in ASPR and ASDR. This suggests that the current male infertility strategy in China is playing a positive role. It should be noted that the BAPC model is based on historical data and current trends, and does not fully account for potential changes in new medical technologies and policy directions that may influence future trends. Therefore, future studies must fully account for these potential influences on trends.

Despite using the latest GBD 2021 database and a variety of statistical models, our study has some limitations. First, the GBD database provides estimates rather than actual data, which may introduce bias, particularly in regions with limited data. Second, the analysis focuses only on male infertility, without considering female factors, which are essential for a comprehensive understanding of infertility. Excluding female factors may limit the accuracy of the study. Third, the lack of regional and urban-rural data limits our ability to fully account for the potential impact of socioeconomic and healthcare differences within China. Fourth, although the GBD database includes subcategories of male infertility, such as azoospermia and drug-induced infertility, the aggregated nature of the data prevents a detailed analysis of these subcategories. Finally, screening and fertility intentions may vary by age group, potentially leading to under-reporting of infertility cases in older men.

These limitations are acknowledged, and future research should address these gaps by incorporating more detailed regional data, including both male and female factors in infertility studies, conducting separate analyses of infertility subcategories where possible, and exploring the influence of age and fertility screening practices.

## 5 Conclusion

In summary, by analysing the GBD2021 data, we found that the disease burden of male infertility in China generally increased from 1990 to 2021. However, thanks to a number of factors, including China's proactive health policies and effective management, the burden of disease has continued to decrease in the last decade and is projected to continue declining from 2022 to 2036. To sustain this positive trend, it remains essential for China to maintain and strengthen effective management and control of male infertility.

## Data Availability

The original contributions presented in the study are included in the article/Supplementary Material, further inquiries can be directed to the corresponding author.
